# Dynamic changes in neural retinal images during the development of a lamellar macular hole: A case report

**DOI:** 10.1097/MD.0000000000018297

**Published:** 2019-12-10

**Authors:** Yoko Ozawa, Hajime Shinoda, Norihiro Nagai, Kazuo Tsubota

**Affiliations:** a Laboratory of Retinal Cell Biology; b Department of Ophthalmology, Keio University, School of Medicine, Shinjukuku, Tokyo, Japan.

**Keywords:** lamellar macular hole, macula, morphology, neurodegeneration, OCT imaging, retina

## Abstract

**Rationale:**

Lamellar macular hole (LMH) comprises a partial-thickness defect of the macula, the central part of the neural retina. Classification of LMHs into degenerative and tractional types has been proposed based on macular morphology observed on optical coherence tomography (OCT) imaging. Although LMHs are assumed to develop from aborted full-thickness macular holes, the clinical course of LMH development is not fully understood.

**Patient concerns:**

A 67-year-old man noticed slight changes in his central vision, and exhibited gradual change in macular morphology.

**Diagnosis:**

The patient was first diagnosed with macular hole, which closed spontaneously. He then developed an LMH.

**Interventions:**

The patient was placed under observation.

**Outcomes:**

The patient first exhibited a macular hole, which resolved spontaneously. However, the tractional force at the surface of the macula became more severe, thereby causing retinoschisis-like appearance, which is the characteristic finding of a tractional LMH. The same eye then developed a lamellar hole-associated epiretinal proliferation (LHEP)-like structure, which is often observed in a degenerative LMH; importantly, the retinoschisis-like appearance persisted. Finally, the macula formed a typical degenerative LMH, characterized by intraretinal cavitation with persistent LHEP and disappearance of the retinoschisis-like structure.

**Lessons:**

The present report shows dynamic changes of neural retinal morphology during development of an LMH: a tractional LMH developed initially; it then transformed into a degenerative LMH, which comprises a neurodegenerative disease. The findings in this report may help to understand the pathogenesis of LMHs and to elucidate the neurodegenerative disease process in various fields.

## 1 Introduction

Recent progress in fundus imaging has enabled the determination of a more precise pathogenesis of macular diseases in which the macula, the central part of the retina, is affected, and thus results in central visual disorders. One such disease is lamellar macular hole (LMH), originally described by Gass.^[1]^ LMH is defined as a partial-thickness neural defect of the macula.^[2]^ Classification of LMHs into tractional and degenerative types has been proposed based on macular morphology observed on optical coherence tomography (OCT) imaging^[3]^; the 2 types are respectively characterized by retinoschisis-like appearance and intraretinal cavitation, which is often associated with lamellar hole-associated epiretinal proliferation (LHEP). Although LMHs are assumed to develop from aborted full-thickness macular holes,^[4,5]^ the clinical course of LMH development is not fully understood. Herein, we report dynamic changes in macular morphology visualized by longitudinal OCT imaging in a patient who eventually developed an LMH.

## 2 Case presentation

**Figure 1:**
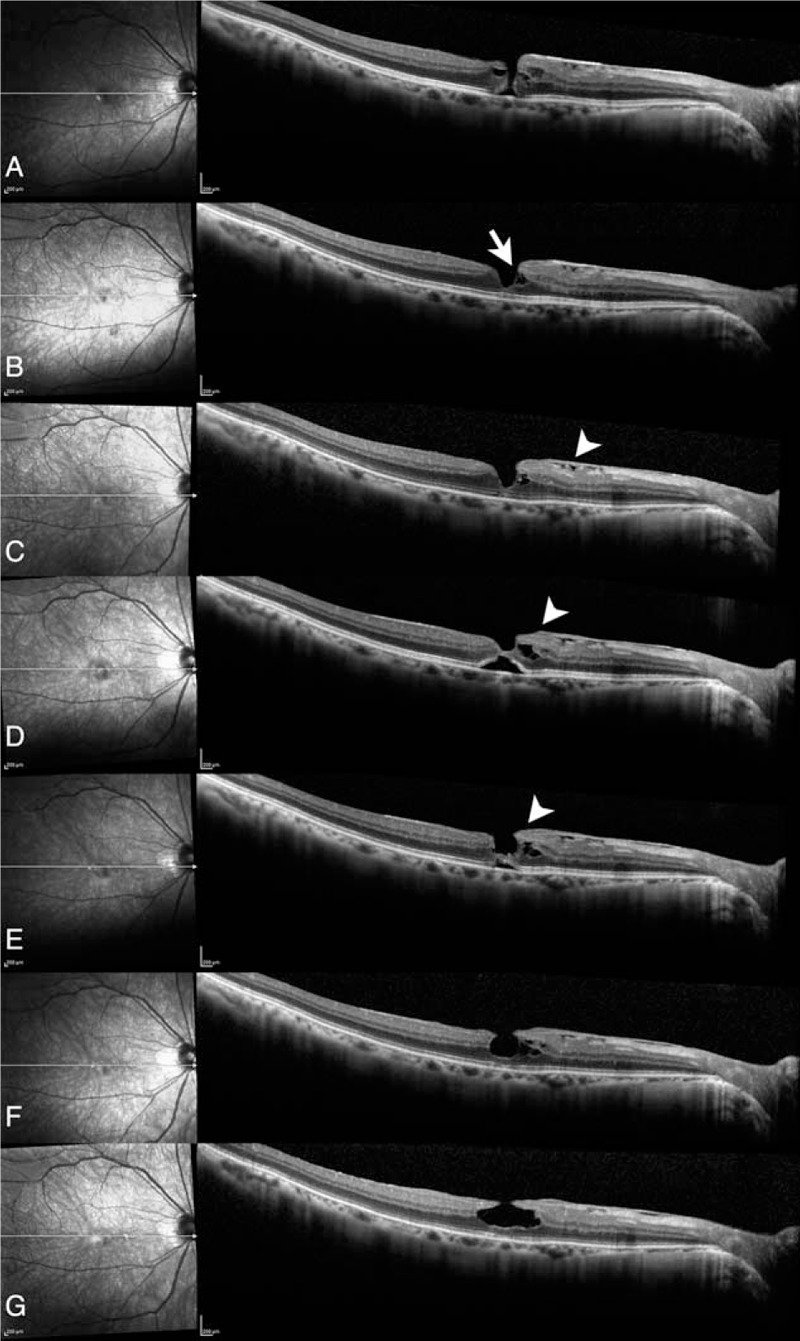
Optical coherence tomography images during the gradual development of a lamellar macular hole. A, In October 2012, optical coherence tomography (OCT) imaging demonstrated a macular hole with cystoid change at its edge in the inner retinal layer and separation of the layer at the outer plexiform layer (OPL). B, In June 2013, the macular hole appeared to have been closed spontaneously, whereas OPL separation (arrow) persisted. C, In August 2013, the epiretinal membrane (arrowhead) became more evident. D, In April 2015, subfoveal detachment developed with enlargement of OPL separation to assume a sharp-edged retinoschisis-like appearance. In addition, a lamellar hole-associated epiretinal proliferation (LHEP)-like structure developed (arrowhead). E, In August 2015, the detachment gradually reduced, whereas the retinoschisis-like appearance persisted. The LHEP-like structure (arrowhead) seemed to be connected to the bottom of the fovea. F, In February 2016, the foveal detachment disappeared. G, In March 2018, OCT imaging showed a typical degenerative LMH with a foveal bump, LHEP, and a disrupted ellipsoid zone. However, the retinoschisis-like appearance had disappeared. The best-corrected visual acuity (BCVA) at each time point in respective decimal score and LogMAR are as follows: 0.9 and −0.046 in October 2012, 1.0 and 0.000 in June 2013, 1.2 and −0.079 in August 2013, 0.8 and 0.097 in April 2015, 0.8 and 0.097 in August 2015, and 0.9 and −0.046 in February 2016, 0.9 and −0.046 in March 2018.

In October 2012, a 67-year-old Japanese man was referred to the Vitreo-Retina Surgical Division Clinic, Department of Ophthalmology, Keio University Hospital. The local clinic had observed a full-thickness macular hole in his right eye. His best-corrected visual acuity (BCVA) was 0.9 in decimal score (−0.046 LogMAR). The OCT image showed a macular hole with cystoid change at its edge in the inner retinal layer and separation of the outer plexiform layer (OPL), where synapses exist between photoreceptor cells and secondary neurons that are bipolar cells (Fig. 1A). Because the patient's symptoms were unclear and BCVA was relatively good, we selected observation without active treatment. At a follow-up visit in June 2013, the macular hole appeared to have closed spontaneously, although OPL separation persisted and retinoschisis-like appearance was observed (Fig. 1B). At subsequent visits, OCT images showed dynamic changes. In August 2013, the patient's epiretinal membrane (ERM) became more evident; the ERM comprises extracellular matrix and causes a tractional force at the retinal surface (Fig. 1C). In April 2015, subfoveal detachment developed with enlargement of the pre-existing OPL separation that had a sharp-edged retinoschisis-like appearance; an LHEP-like structure was also observed (Fig. 1D). In August 2015, the detachment gradually reduced, whereas the retinoschisis-like appearance persisted (Fig. 1E). The LHEP-like structure seemed to be connected to the bottom of the fovea. In February 2016, the foveal detachment disappeared (Fig. 1F). The patient was referred back to the local clinic because his BCVA was 0.9 in decimal score (−0.046 LogMAR); thus, no surgery was planned. In March 2018, he was referred to us again for cataract surgery. Preoperative OCT examination showed a typical degenerative LMH with intraretinal cavitation (Fig. 1G). The OCT image showed a foveal bump, LHEP, and a disrupted ellipsoid zone in the photoreceptor layer. However, the clear retinoschisis-like appearance had disappeared. The patient's BCVA was 0.9 in decimal score (−0.046 LogMAR). After cataract surgery, his BCVA recovered to 1.2 in decimal score (−0.079 LogMAR), which is maintained to date; the LMH has not shown further changes (data not shown).

## 3 Discussion

Govetto et al^[3]^ proposed that the 2 subtypes of LMHs, tractional and degenerative, represent different pathologic conditions. The tractional LMHs have a characteristic schisis-like separation between the outer plexiform and outer nuclear layer.^[6]^ Hirano et al^[7]^ reported that the retinal fold caused by tractional force at the vitreoretinal interface is often observed in tractional LMHs on the en face images of the OCT. In contrast, degenerative LMHs do not demonstrate splitting of the retina along any definite cleavage plane, and can affect all retinal layers.^[3]^ Govetto et al^[3]^ speculated that the degenerative LMHs may have a slow, chronic process of causing loss of retinal tissue. However, they also reported that 10% of LMH patients had a mixed lesion with degenerative and tractional features, and older patients had degenerative LMHs.^[3]^ Notably, our patient first exhibited a retinoschisis-like appearance, which represents tractional LMH; subsequently, the same retina dynamically changed to degenerative LMH. The LHEP-like structure is a thick homogenous layer of material with medium reflectivity at the epiretinal surface on OCT images, different from ERM which involves tractional properties,^[8]^ and supposed to be one of the characteristic findings of degenerative LMHs.^[3]^ However, in the current case, it was present when the eye exhibited tractional LMH, before the development of degenerative LMH. Therefore, some degenerative LMHs may have exhibited a tractional stage. In this case, the tractional force of the ERM may have separated the macular tissue and induced Müller glial migration to form LHEP; a histological report showed that Müller glial cells were involved in the LHEP.^[9]^ Given that Müller glial cells migrated out from the retina, the retinal tissue then formed a degenerative LMH. Although the etiology and developmental course of LMHs may not have a single explanation, the present report, which shows dynamic changes of neural retinal morphology during development of an LMH, may help to understand the pathogenesis of this neurodegenerative disease. In addition, the findings in this report may also help to elucidate the neurodegenerative disease process in various fields.

This study followed the tenets of the Declaration of Helsinki, and was approved by the Ethics Committee of the Keio University School of Medicine. Informed consent was obtained from the patient for publication of this case report.

## Acknowledgments

The authors thank their colleagues and the paramedical staff in the Department of Ophthalmology, Keio University Hospital (Tokyo, Japan) for assisting them.

## Author contributions

**Conceptualization:** Yoko Ozawa.

**Data curation:** Yoko Ozawa.

**Writing – original draft:** Yoko Ozawa.

**Writing – review & editing:** Hajime Shinoda, Norihiro Nagai, Kazuo Tsubota.

Yoko Ozawa orcid: 0000-0003-4797-5705.
